# Pungenin Promotes Hair Growth in Mouse Models of Androgenic Alopecia Through Transcriptional Regulation of the PI3K/AKT/mTOR Axis

**DOI:** 10.3390/molecules31162864

**Published:** 2026-08-17

**Authors:** Wencai Zhai, Jun Yang, Lei Zhang, Zikai Lin, Wendi Xie, Shaohong Wen, Gang Li

**Affiliations:** College of Life Sciences, Yantai University, Yantai 264003, China; zhaiwencai@163.com (W.Z.); yangjun010218@163.com (J.Y.); zhanglei982024@163.com (L.Z.); lzk1215741582@163.com (Z.L.); wendi_xie@126.com (W.X.); wenshaohong1@163.com (S.W.)

**Keywords:** pungenin, *Picea wilsonii* Mast, androgenetic alopecia, hair follicle cells, PI3K/AKT/FoxO/mTOR signaling

## Abstract

Androgenetic alopecia (AGA) is the most common form of hair loss, characterized by the progressive miniaturization of hair follicles driven by dihydrotestosterone (DHT)-mediated androgen receptor signaling. Current pharmacotherapies remain limited in efficacy and are often associated with undesirable side effects. Pungenin, a phenolic glucoside isolated from *Picea wilsonii* Mast., was investigated for its therapeutic efficacy in DHT-induced AGA model mice in this study. In primary mouse hair follicle cells exposed to DHT, pungenin mitigated cellular injury, restored normal morphology and viability, and significantly reduced the expression of type II 5α-reductase (*Srd5α2*). Topical treatment with pungenin markedly accelerated hair regeneration, as demonstrated by histological analysis and serum biochemical measurements. Transcriptomic profiling combined with network pharmacology suggested that the protective effects of pungenin are associated with transcriptional regulation of the PI3K/AKT/FoxO/mTOR axis, identifying 25 putative therapeutic targets, with qPCR further confirming the altered expression of key genes within this network. Collectively, these findings demonstrate that pungenin protects hair follicles against DHT-induced injury through suppression of *Srd5α2* and transcriptional modulation of the PI3K/AKT/mTOR signaling axis, supporting further preclinical evaluation of pungenin as a potential candidate for AGA management.

## 1. Introduction

Androgenetic alopecia (AGA) is the most common form of hair loss, affecting up to 80% of men and 50% of women by the age of 70, with a significant global burden [1]. Beyond its physical manifestations, AGA frequently causes substantial psychological distress and negatively impacts patients’ quality of life [2,3]. Normal hair growth depends on the orderly progression through the sequential phases of anagen (growth), catagen (regression), and telogen (resting), which together preserve hair follicle homeostasis [4,5]. In patients with AGA, the hallmark pathological alteration is progressive shortening of the anagen phase, ultimately resulting in follicular miniaturization. During this process, thick terminal hairs are gradually replaced by fine vellus hairs, accompanied by progressive follicular atrophy and eventual hair loss [6,7]. The etiology of AGA involves interactions among genetic predisposition, androgen signaling, perifollicular microinflammation, impaired microcirculation, and metabolic abnormalities [8,9,10].

Of these pathogenic mechanisms, androgen signaling is regarded as the principal driver of disease progression. Type II 5α-reductase (*Srd5α2*) catalyzes the conversion of testosterone into the more biologically active androgen dihydrotestosterone (DHT). DHT subsequently binds androgen receptors expressed by dermal papilla cells (DPCs), disrupting normal regulation of the hair cycle through altered cytokine expression. These molecular changes promote follicular miniaturization and accelerate the transition from the anagen to the resting phase [11,12,13]. Consequently, finasteride, a selective inhibitor of *Srd5α2*, has received FDA approval for the treatment of male AGA [14,15]. Nevertheless, its widespread clinical application is limited by adverse reactions, including dizziness, headache, anxiety, and cognitive impairment [16,17]. Furthermore, symptoms may persist after discontinuing therapy, described as post-finasteride syndrome [18,19]. These limitations underscore the need to develop alternative therapies with improved efficacy and safety.

In addition to androgen signaling, accumulating evidence has highlighted the PI3K/AKT/mTOR pathway as a critical regulator of hair follicle dynamics. This signaling axis modulates hair follicle stem cell proliferation, survival, and anagen maintenance. Dysregulation of this pathway has been implicated in AGA pathogenesis, and its activation has been shown to promote hair cycle progression and hair growth [20]. These findings suggest that therapeutic agents capable of modulating this pathway may hold promise for AGA treatment.

Natural products have been established as attractive sources of novel therapeutics for hair loss because of their structural diversity and ability to regulate multiple biological targets simultaneously [21,22]. Species of the genus Picea have attracted considerable pharmacological interest. Extracts from Picea pungens, Picea orientalis, and Picea glauca have demonstrated antioxidant, anti-inflammatory, antimicrobial, and lipid-lowering activities [23,24]. By comparison, *Picea wilsonii* Mast., a conifer native to China, has received relatively little scientific attention. In traditional Chinese medicine, this species has historically been prescribed for the treatment of palpitations, insomnia, restlessness, dizziness, and headache, and promotes blood circulation, relieve pain, and produce sedative effects [25]. Despite its long-standing traditional use, comprehensive investigations of its phytochemical composition and pharmacological properties remain limited, leaving its therapeutic potential largely unexplored.

We previously isolated the phenolic glucoside pungenin from the branches of *Picea wilsonii* Mast. for the first time and demonstrated that this compound exhibits potent inhibitory activity against *Srd5α2* [26]. These findings prompted us to evaluate whether pungenin could protect hair follicles against DHT-induced injury. In the present study, we assessed the hair-growth promoting effects of pungenin in a DHT-induced AGA mouse model and further elucidated the underlying molecular mechanisms through transcriptomic profiling, network pharmacology, and qPCR validation. Given the established role of the PI3K/AKT/mTOR axis in hair follicle cycling and survival [20], we hypothesized that pungenin may exert its protective effects through transcriptional modulation of this signaling network.

Unlike crude botanical extracts such as pea sprout extract, fucoidan, or serenoa repens, which contain complex mixtures of active compounds, pungenin is a structurally defined phenolic glucoside. This chemical purity enables precise mechanistic studies and consistent quality control. Moreover, while most natural anti-AGA agents act through a single pathway—typically *Srd5α2* inhibition or antioxidant activity—pungenin appears to exert its effects through a dual mechanism: direct inhibition of *Srd5α2* and transcriptional regulation of the PI3K/AKT/FoxO/mTOR signaling axis. This dual-targeted profile positions pungenin as a distinctive lead compound for further development in AGA therapy.

## 2. Results

### 2.1. Isolation and Characterization of Primary Hair Follicle Cells

**Figure 1:**
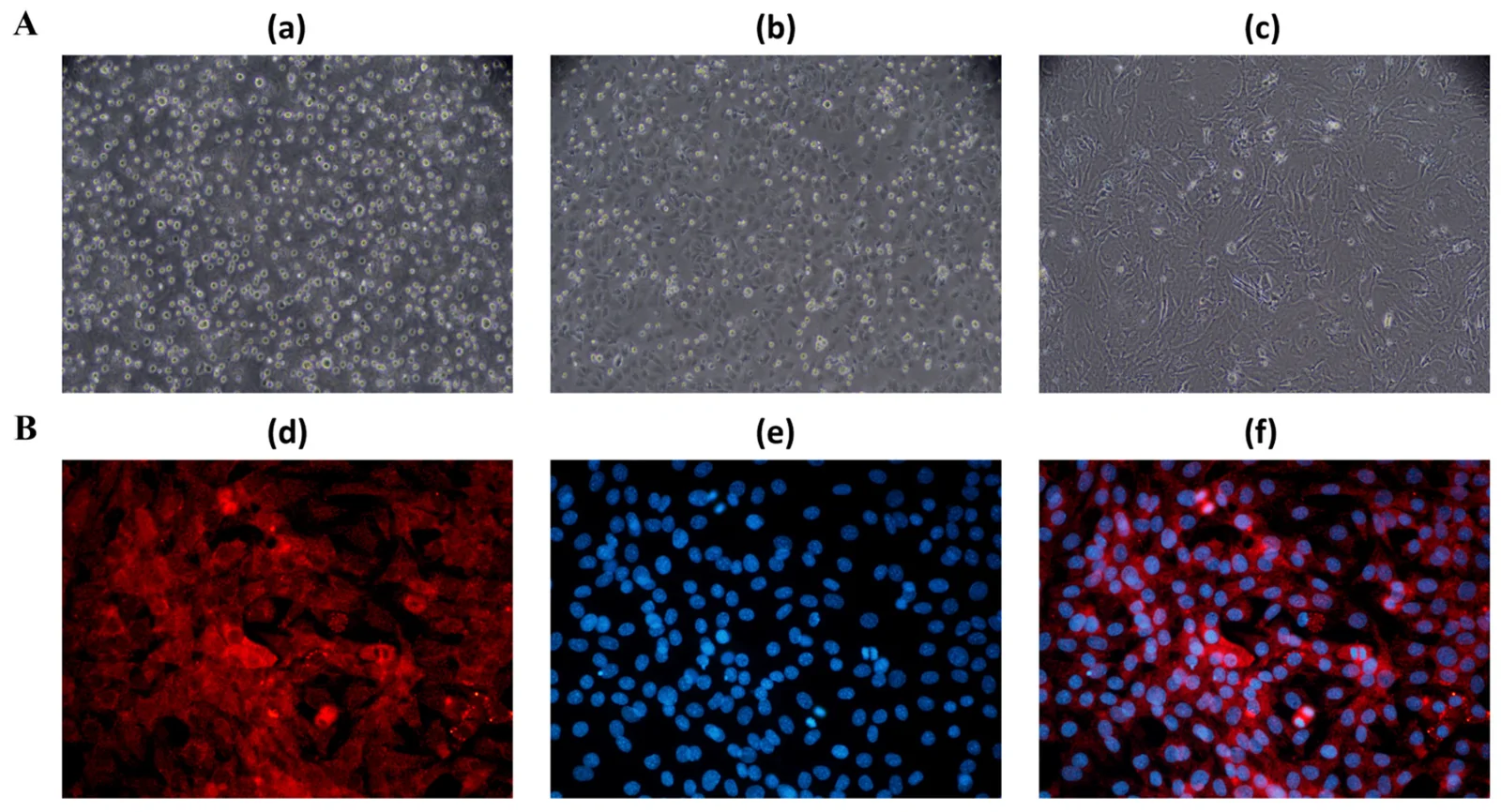
Isolation, culture, and identification of primary mouse hair follicle cells. (**A**) Representative images of cultured primary hair follicle cells (200× magnification): (**a**) immediately after overnight digestion and seeding; (**b**) after 6 h of adhesion; (**c**) after 3 days of culture. (**B**) CK-19 immunofluorescence staining of primary mouse hair follicle cells (400× magnification): (**d**) CK-19; (**e**) DAPI; (**f**) merged CK-19/DAPI image.

Primary mouse hair follicle cells were successfully isolated and cultured in vitro (Figure 1A). Following overnight enzymatic digestion, the cells adhered readily and displayed elongated triangular or polygonal morphologies. After 3 days of culture, the cells proliferated extensively and formed characteristic radial outgrowths with irregular spindle-shaped morphology. Immunofluorescence staining for CK-19 (Figure 1B) confirmed the identity of the isolated cells, with a purity exceeding 90%.

### 2.2. Effects of Pungenin on Hair Follicle Cell Viability and Morphology

**Figure 2:**
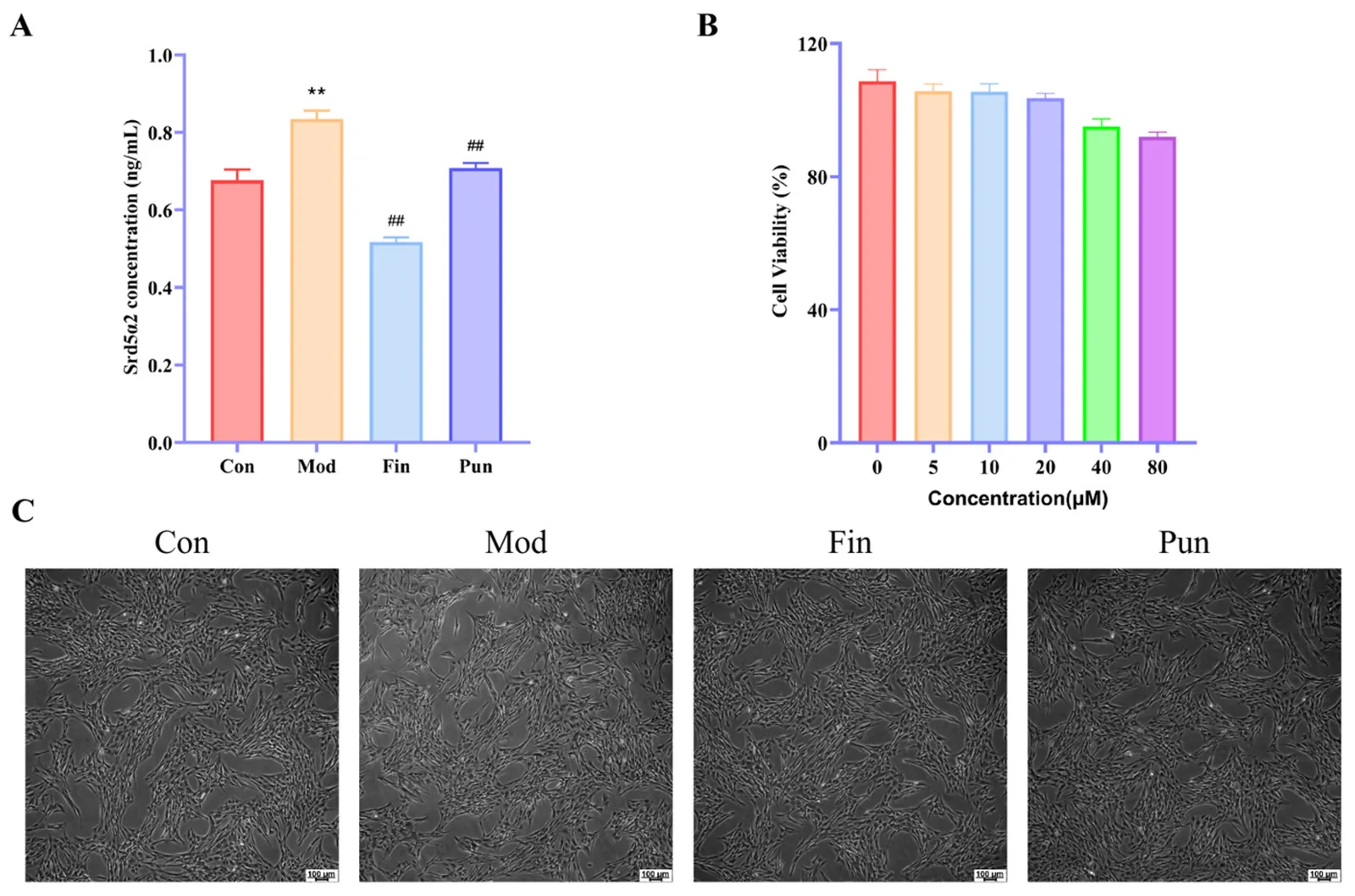
Protective effects of pungenin on primary mouse hair follicle cells. (**A**) Effect of pungenin on *Srd5α2* production; (**B**) effect of pungenin on cell viability; (**C**) representative phase-contrast images of cell morphology (100× magnification). Data are presented as mean ± SD (*n* = 3) based on three independent experiments. ** *p* < 0.01 compared with the control group; ## *p* < 0.01 compared with the model group.

The effects of pungenin on *Srd5α2* production are presented in Figure 2A. SRB assay results (Figure 2B) demonstrated that pungenin concentrations up to 40 μM produced no significant cytotoxicity, whereas concentrations above 80 μM substantially lowered cell viability. Thus, 20 μM was used as the highest non-toxic concentration for further studies. Morphological observations are shown in Figure 2C. Exposure to DHT caused pronounced cellular injury, characterized by cell shrinkage, disruption of cell–cell contacts, and irregular cell morphology. In contrast, treatment with either finasteride or pungenin substantially alleviated these morphological abnormalities, indicating that pungenin effectively protects hair follicle cells against DHT-induced damage.

### 2.3. Transcriptomic Analysis

**Figure 3:**
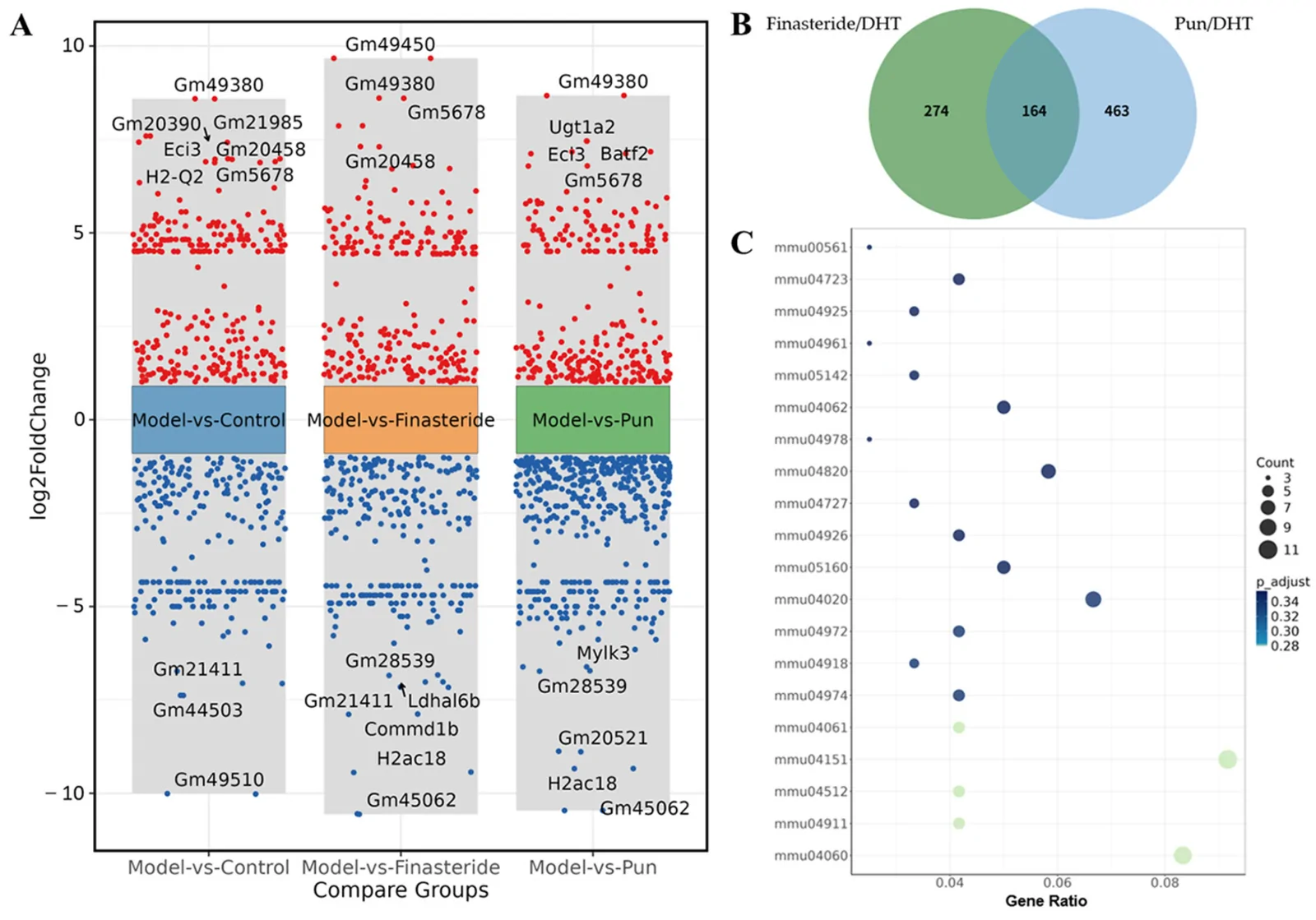
Differential gene expression analysis. (**A**) Multi-group scatter plot comparison; (**B**) Venn diagram showing DEGs in the Finasteride/DHT and Pun/DHT comparisons; (**C**) KEGG enrichment of DEGs identified in the Pun vs. DHT comparison.

Transcriptomic sequencing identified a total of 463 DEGs between the pungenin-treated and DHT-treated groups (Figure 3A,B). KEGG enrichment analysis indicated marked associations of DEGs with pathways involving cytokine–cytokine receptor interactions and PI3K/AKT signaling (Figure 3C), suggesting that modulation of the PI3K/AKT axis may contribute substantially to the biological activity of pungenin.

**Figure 4:**
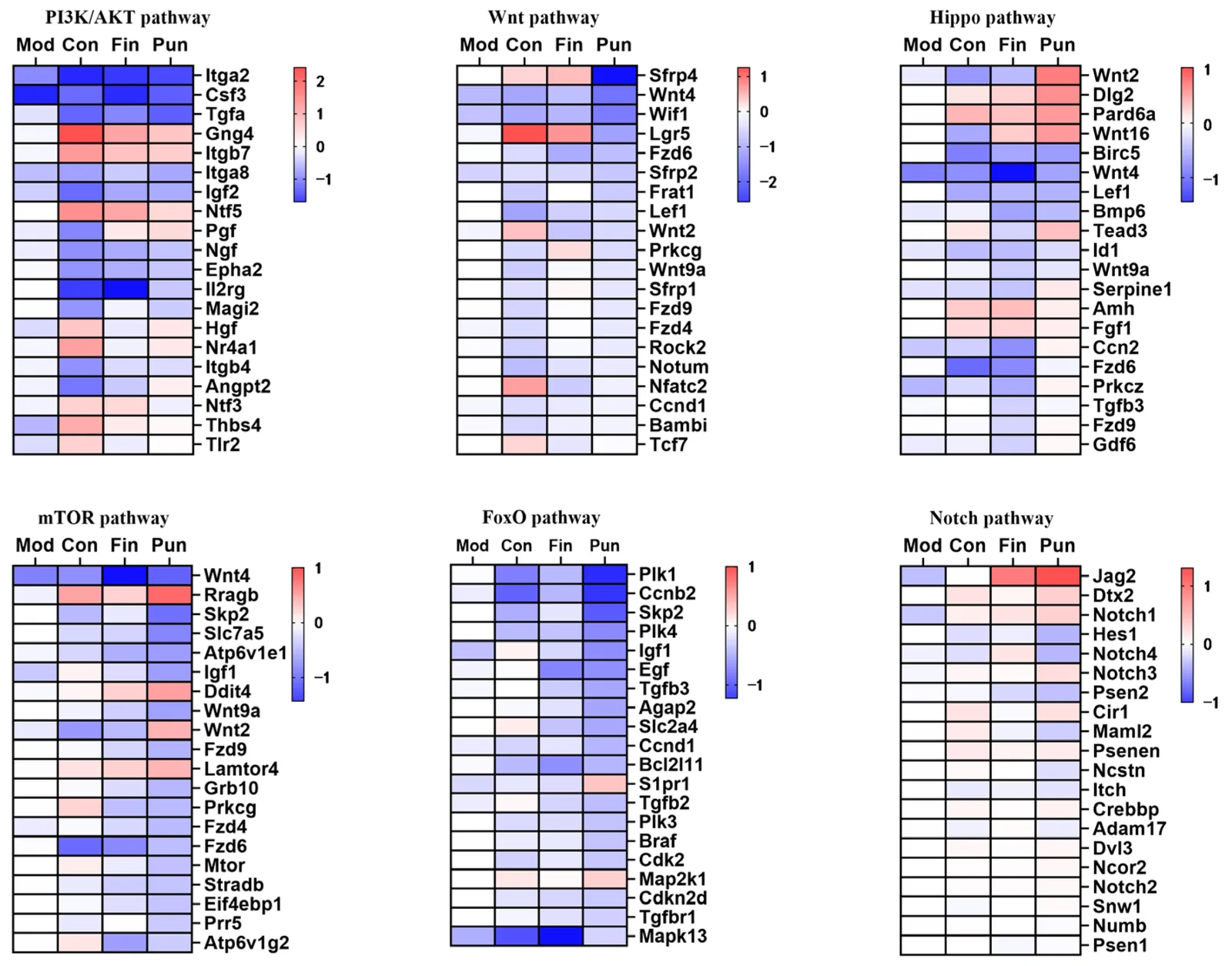
Expression profiles of genes associated with hair follicle development in the Control, Model, Fin, and Pun groups, including genes involved in the PI3K, Wnt, Hippo, mTOR, FoxO, and Notch signaling pathways.

**Figure 5:**
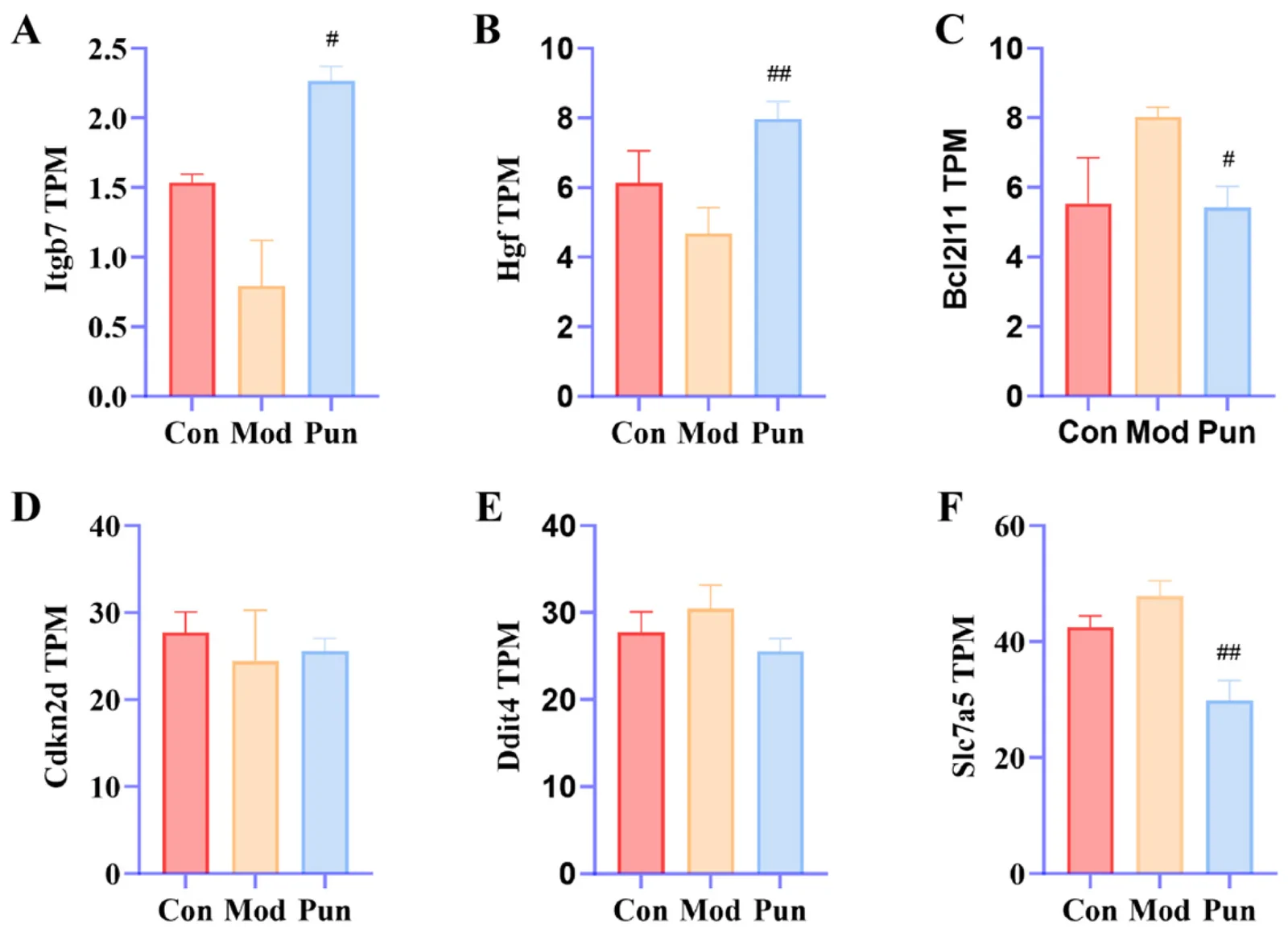
TPM values of representative pathway-related genes. (**A**) TPM values of *Itgb7* in the PI3K/AKT pathway; (**B**) TPM values of *Hgf* in the PI3K/AKT pathway; (**C**) TPM values of *Bcl2l11* in the FoxO pathway; (**D**) TPM values of *Cdkn2d* in the FoxO pathway; (**E**) TPM values of *Ddit4* in the mTOR pathway; (**F**) TPM values of *Slc7a5* in the mTOR pathway. # *p* < 0.05 and ## *p* < 0.01 compared with the model group.

Heatmap analysis of genes associated with hair follicle development (Figure 4) further revealed that pungenin altered the transcriptional profiles of multiple signaling pathways involved in follicle biology, including the PI3K/AKT, Wnt, Hippo, mTOR, FoxO, and Notch pathways. More specifically, pungenin increased the expression of *Itgb7* and *Hgf* within the PI3K/AKT pathway, reduced the levels of the pro-apoptotic gene *Bcl2l11* and the cell cycle inhibitor *Cdkn2d* in the FoxO pathway, and upregulated *Ddit4* while suppressing *Slc7a5* expression in the mTOR pathway (Figure 5). These findings are consistent with the hypothesis that pungenin may promote hair growth, at least in part, through transcriptional regulation of the PI3K/AKT/FoxO/mTOR signaling network.

### 2.4. Effects of Pungenin on mRNA Expression of Mouse Hair Follicle Cells

**Figure 6:**
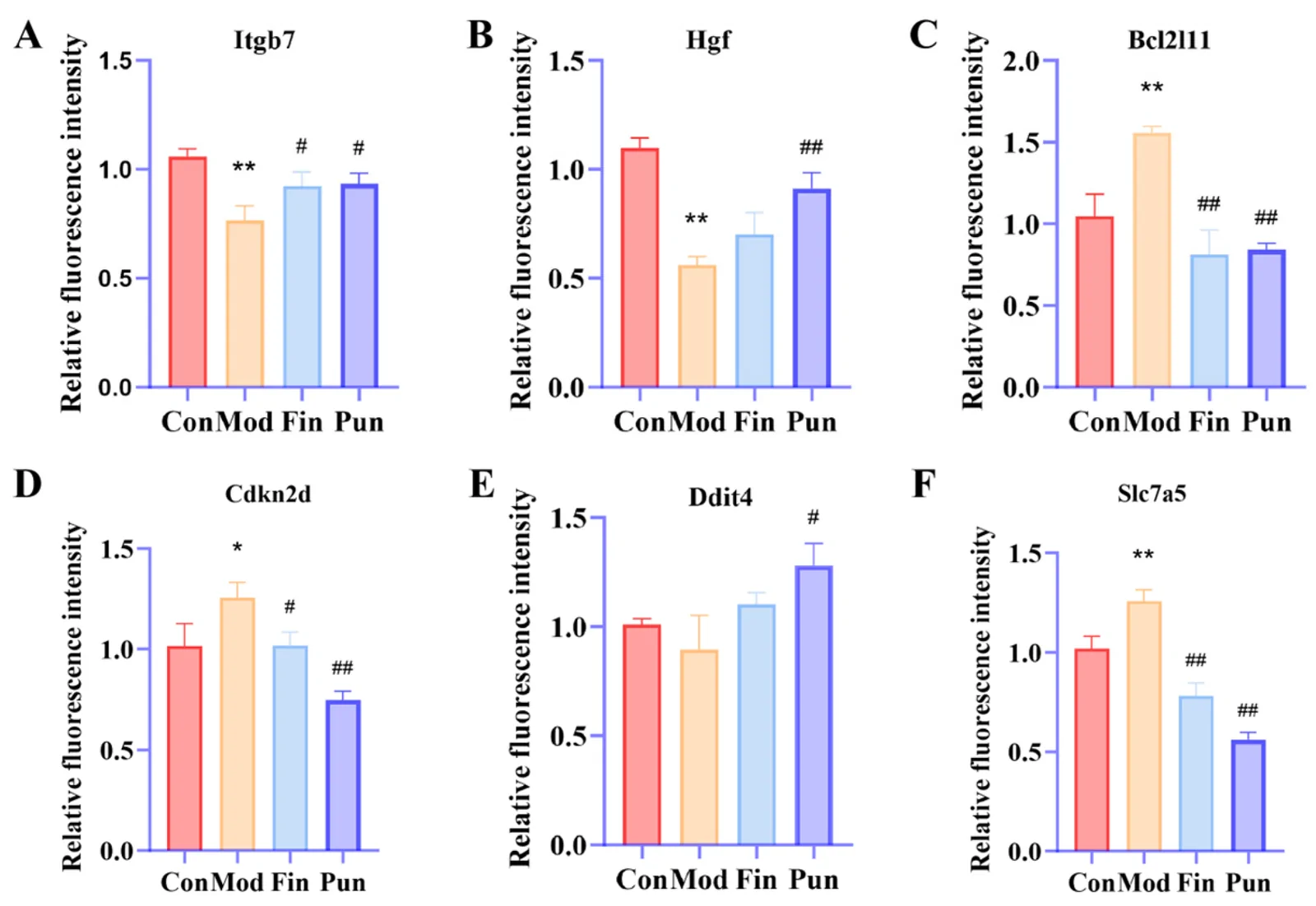
qPCR verification of differentially expressed genes associated with the PI3K/AKT, FoxO, and mTOR axes in hair follicle cells. (**A**) Relative expression of *Itgb7* in the PI3K/AKT pathway; (**B**) Relative expression of *Hgf* in the PI3K/AKT pathway; (**C**) Relative expression of *Bcl2l11* in the FoxO pathway; (**D**) Relative expression of *Cdkn2d* in the FoxO pathway; (**E**) Relative expression of *Ddit4* in the mTOR pathway; (**F**) Relative expression of *Slc7a5* in the mTOR pathway. Data are presented as mean ± SD (*n* = 3) based on three independent experiments. * *p* < 0.05 and ** *p* < 0.01 compared with the control group; # *p* < 0.05 and ## *p* < 0.01 compared with the model group.

qPCR analysis confirmed the reliability of the transcriptomic data (Figure 6). Relative to the model group, pungenin treatment led to markedly increased mRNA levels of *Itgb7*, *Hgf*, and *Ddit4*, while substantially reducing the expression of *Bcl2l11*, *Cdkn2d* and *Slc7a5*. These findings are fully consistent with the RNA-sequencing results.

### 2.5. Assessment of Mice Plasma Biochemical Indicators

**Figure 7:**
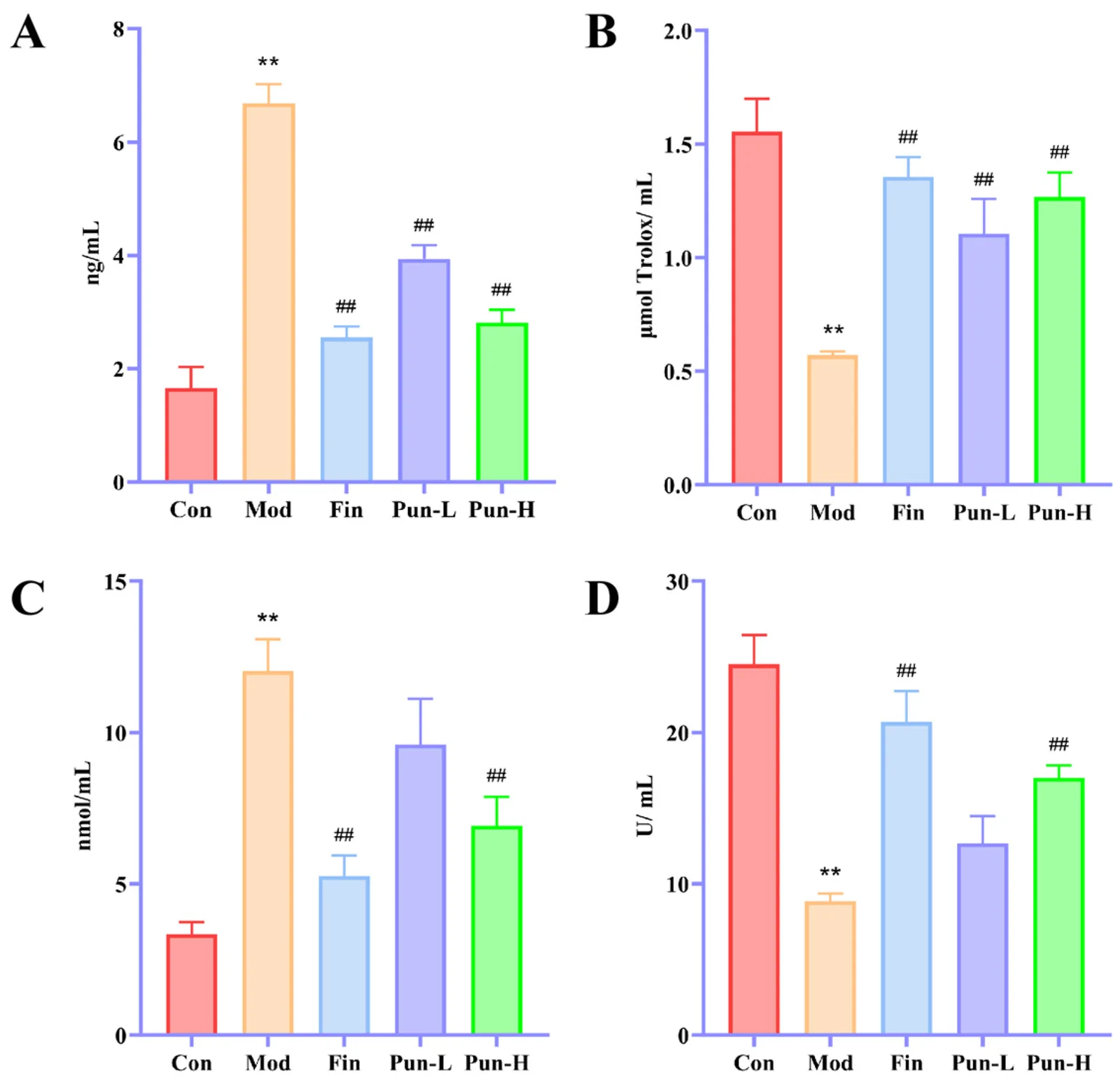
Biochemical analysis of mouse plasma. (**A**) *Srd5α2* concentration; (**B**) T-AOC; (**C**) MDA concentration; (**D**) SOD activity. Data are presented as mean ± SD (*n* = 5) based on three independent experiments. ** *p* < 0.01 compared with the control group; ## *p* < 0.01 compared with the model group.

As illustrated in Figure 7A, serum *Srd5α2* concentrations were markedly increased in the model group relative to the control group, confirming successful establishment of the androgenetic alopecia model. Treatment with either finasteride or pungenin significantly reduced *Srd5α2* levels, indicating that pungenin suppresses *Srd5α2* production. Evaluation of oxidative stress biomarkers (Figure 7B–D) further demonstrated that pungenin significantly elevated serum T-AOC and SOD activity while reducing MDA levels compared with the model group. These findings indicate that pungenin mitigates oxidative stress in a dose-related manner.

### 2.6. Histological Analysis of the Dorsal Skin

**Figure 8:**
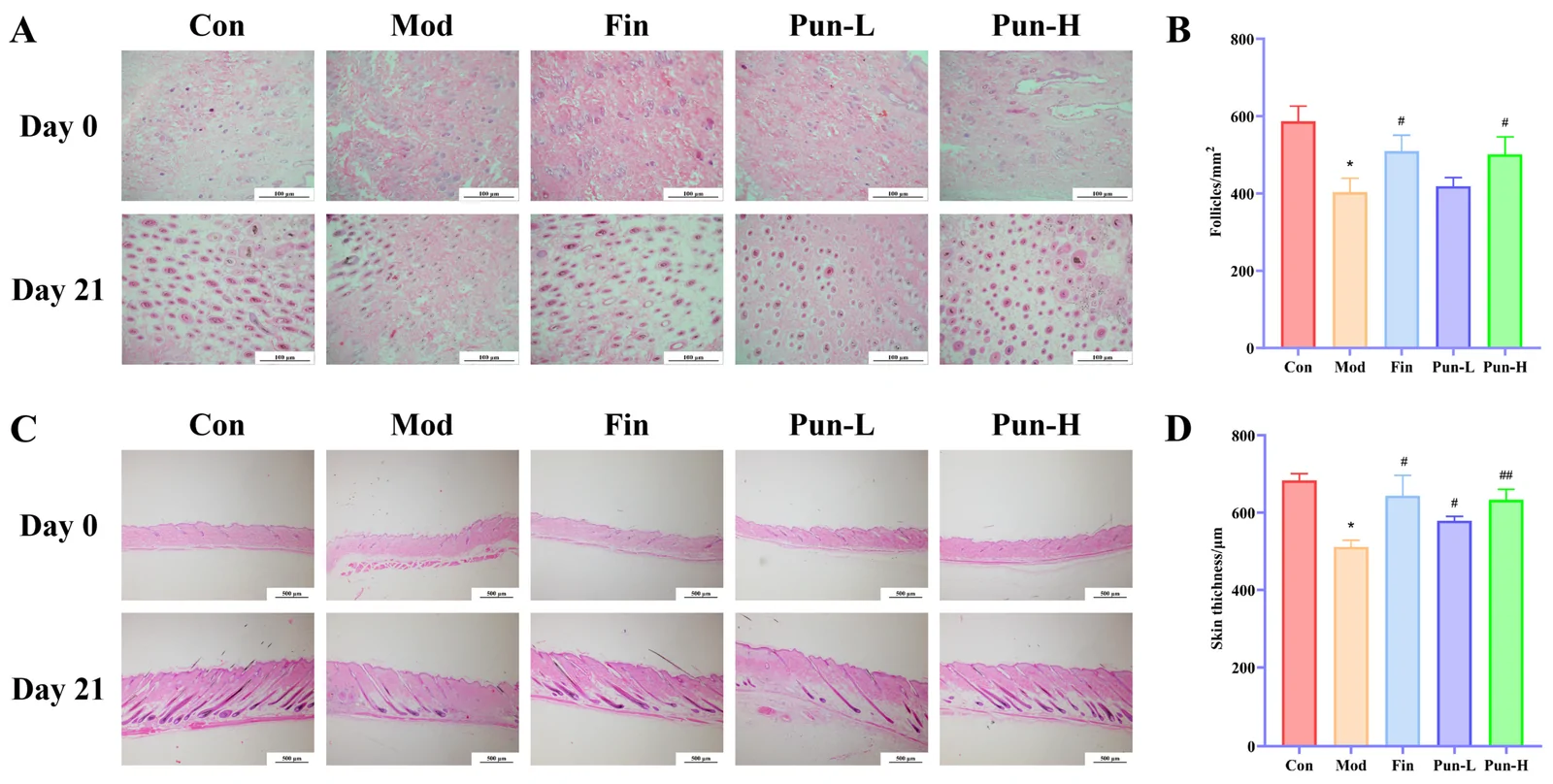
Hematoxylin and eosin (H&E) staining of dorsal skin sections. (**A**) Transverse sections, scale bar = 100 µm; (**B**) Quantitative analysis of hair follicle density; (**C**) Longitudinal sections, scale bar = 500 µm; (**D**) Quantitative analysis of the thickness of dorsal skin. Data are presented as mean ± SD (*n* = 5) based on three independent experiments. * *p* < 0.05 compared with the control group; # *p* < 0.05 and ## *p* < 0.01 compared with the model group.

Histopathological evaluation of dorsal skin sections is presented in Figure 8. Examination of transverse sections (Figure 8A) showed no obvious differences in hair follicle density among the groups immediately after depilation (day 0). By day 21, complete maturation of hair follicles was observed in all treatment groups, whereas follicular development remained markedly impaired in the model group. Pungenin restored both follicle density and structural integrity in a dose-related manner, with the high-dose group exhibiting histological features comparable to those of the finasteride-treated mice. Analysis of longitudinal sections (Figure 8C) likewise demonstrated similar skin thickness across all groups at baseline. At the end of the experiment, robust hair regeneration was evident in every treatment group except the model group. Moreover, mice receiving the higher dose of pungenin exhibited greater recovery of skin architecture and hair growth than those treated with the lower dose.

At the end of the experiment (day 21), compared with the control group, the model group exhibited a significant reduction in hair follicle density (per mm^2^) and dermal thickness (Figure 8B,D), both of which were markedly restored following finasteride intervention. In the Pun-H group, hair follicle density in the dorsal skin was significantly increased, and the recovery of dermal thickness was more pronounced. The Pun-H group showed histological improvements that were similar in magnitude to those observed in the finasteride-treated group, although no direct statistical comparison was performed between these two groups. Throughout the experiment, no local irritation, weight loss, or abnormal behavior was observed in any group of the mice, indicating that pungenin was well tolerated at the tested concentrations.

### 2.7. Effects of Pungenin on mRNA Expression of Mice Dorsal Tissues

**Figure 9:**
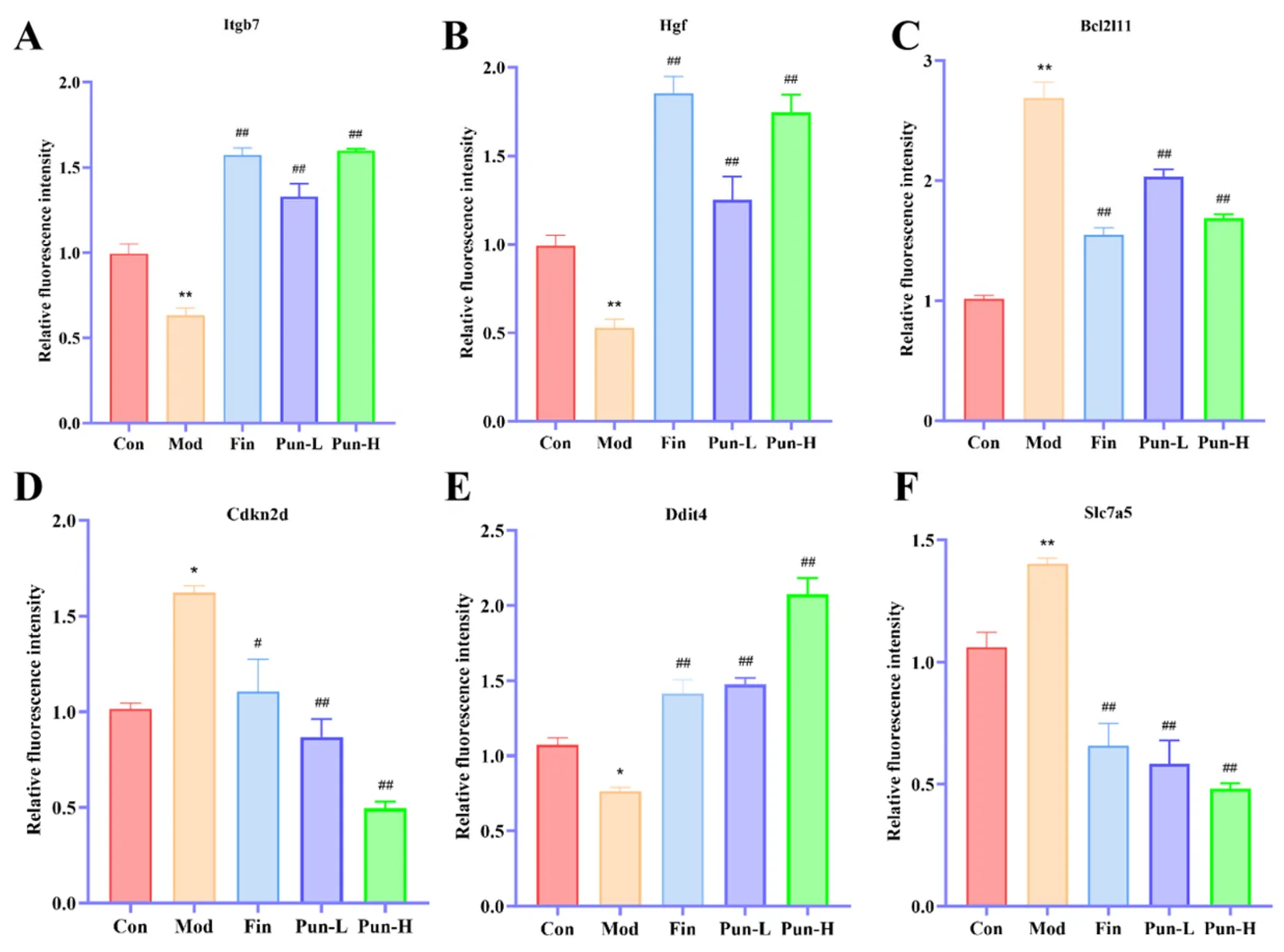
qPCR verification of differentially expressed genes associated with the PI3K/AKT, FoxO, and mTOR axes in mice dorsal tissues. (**A**) Relative expression of *Itgb7* in the PI3K/AKT pathway; (**B**) Relative expression of *Hgf* in the PI3K/AKT pathway; (**C**) Relative expression of *Bcl2l11* in the FoxO pathway; (**D**) Relative expression of *Cdkn2d* in the FoxO pathway; (**E**) Relative expression of *Ddit4* in the mTOR pathway; (**F**) Relative expression of *Slc7a5* in the mTOR pathway. Data are presented as mean ± SD (*n* = 3) based on three independent experiments. * *p* < 0.05 and ** *p* < 0.01 compared with the control group; # *p* < 0.05 and ## *p* < 0.01 compared with the model group.

To confirm the effect of pungenin applied topically on hair growth in mice, the relevant mRNA expression levels (*Itgb7*, *Hgf*, *Ddit4*, *Bcl2l11*, *Cdkn2d*, and *Slc7a5*) were measured by qPCR (Figure 9). These results corroborate the qPCR findings from mouse hair follicle cells.

### 2.8. Network Pharmacology Results

**Figure 10:**
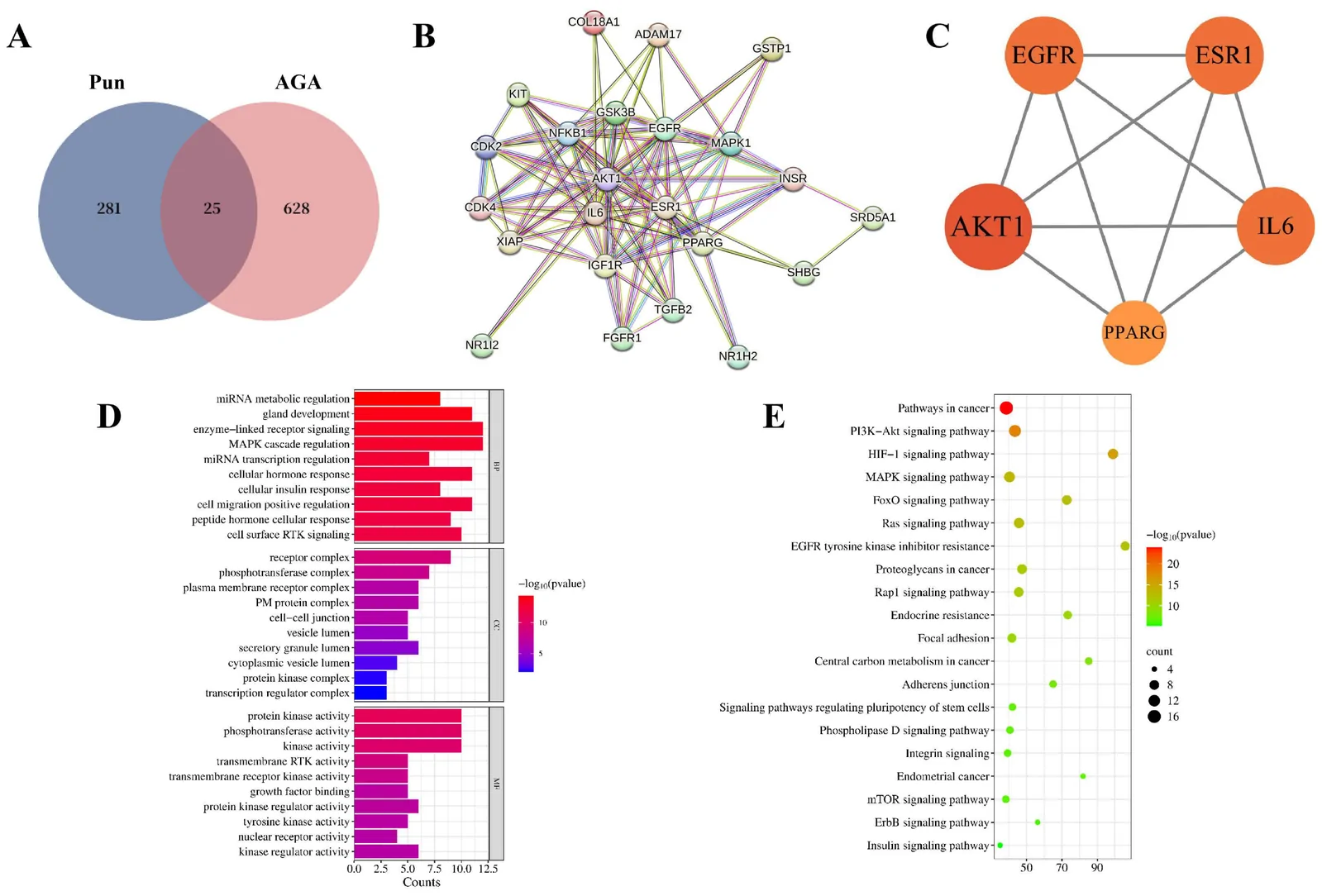
Network pharmacology analysis. (**A**) Venn diagram showing overlapping targets between pungenin and AGA; (**B**) PPI network of common therapeutic targets: Different colored lines indicate the types of supporting evidence: purple for experimental evidence, light blue for curated database evidence, yellow for text mining, black for co-expression, blue for gene co-occurrence, green for gene neighborhood, and red for gene fusion; (**C**) Hub target genes identified by CytoHubba; (**D**) GO enrichment; (**E**) KEGG pathway enrichment.

Network pharmacology identified 306 putative targets of pungenin and 653 genes associated with AGA. Intersection analysis revealed 25 shared targets (Figure 10A), which were subsequently used to construct a PPI network (Figure 10B). Hub gene analysis using CytoHubba identified AKT1, EGFR, ESR1, IL6, and PPARG as the principal candidate targets mediating the therapeutic effects of pungenin (Figure 10C). GO and KEGG pathway enrichment analyses (Figure 10D,E) demonstrated that these common targets were mainly enriched in the PI3K/AKT, mTOR, and FoxO axes, providing independent support for the transcriptomic findings.

In addition to regulating cell proliferation and survival, the PI3K/AKT pathway is closely integrated with cellular redox regulation. Previous studies have shown that reactive oxygen species (ROS) can influence PI3K/AKT signaling through oxidative modification of upstream phosphatases, particularly PTEN [27]. Recent evidence has shown that natural products can promote hair regeneration through combined anti-oxidative and growth factor mechanisms, and the AKT/mTORC1 axis has emerged as a central regulator of hair follicle stem cell activation and cycling [28,29].

In the present study, pungenin significantly decreased serum MDA levels while increasing T-AOC and SOD activity, indicating a substantial reduction in DHT-induced oxidative stress. By alleviating oxidative damage, pungenin may preserve AKT phosphorylation and maintain downstream transcriptional activity, as excessive oxidative stress is known to impair PI3K/AKT signaling through PTEN oxidation. The predicted hub targets also support this interpretation. Notably, the predicted hub targets, including AKT1, EGFR, IL6, and PPARG, are closely associated with AGA-related pathways. Among these, AKT1 is a central node in the PI3K/AKT signaling pathway, which was also identified as a significantly enriched pathway in our transcriptomic analysis (Figure 3C), supporting the consistency between the network pharmacology predictions and the experimental data. Therefore, the hair growth-promoting effects of pungenin likely result from two complementary mechanisms: direct transcriptional regulation of the PI3K/AKT/FoxO/mTOR axis and indirect preservation of pathway activity through attenuation of oxidative stress. Together, these actions enhance the survival and proliferative capacity of hair follicle cells.

## 3. Discussion

In the present study, we demonstrated that pungenin, a phenolic glucoside isolated from *Picea wilsonii* Mast., promotes hair regeneration in a DHT-induced AGA mouse model. Our findings indicate that the protective effects of pungenin involve multiple complementary mechanisms, including suppression of *Srd5α2* expression, attenuation of oxidative stress, and transcriptional regulation of the PI3K/AKT/FoxO/mTOR signaling axis.

Among these, the inhibition of *Srd5α2* is particularly noteworthy, as this enzyme catalyzes the conversion of testosterone to DHT and is a well-established target for AGA treatment. Our results showed that pungenin significantly reduced *Srd5α2* levels in both cultured hair follicle cells and mouse serum, suggesting a direct inhibitory effect comparable to that of finasteride, though direct potency comparisons were not performed. This finding positions pungenin as a promising lead compound for further optimization.

Transcriptomic profiling and network pharmacology consistently pointed to the PI3K/AKT/FoxO/mTOR axis as a key pathway affected by pungenin. Specifically, pungenin upregulated the expression of *Itgb7* and *Hgf* (PI3K/AKT), downregulated the pro-apoptotic gene *Bcl2l11* and cell-cycle inhibitor *Cdkn2d* (FoxO), and modulated *Ddit4* and *Slc7a5* (mTOR). These transcriptional changes are consistent with enhanced cell survival, reduced apoptosis, and improved proliferative capacity of hair follicle cells. The convergence of transcriptomic and network pharmacology data strengthens the validity of these findings and supports the hypothesis that pungenin acts, at least in part, through transcriptional modulation of this signaling network.

In addition to its effects on gene expression, pungenin also improved serum antioxidant parameters, as evidenced by elevated T-AOC and SOD activity and reduced MDA levels. This antioxidative effect may contribute to the preservation of AKT phosphorylation by reducing oxidative stress-mediated PTEN oxidation, thereby indirectly supporting PI3K/AKT pathway activity.

The consistency of our data across different experimental platforms supports the biological relevance of pungenin’s effects. In cultured ORS cells, pungenin upregulated *Itgb7*, *Hgf*, and *Ddit4* while downregulating *Bcl2l11*, *Cdkn2d*, and *Slc7a5*, indicating enhanced survival and reduced apoptosis. These transcriptional changes are mirrored in dorsal skin tissues from pungenin-treated mice, where similar expression patterns were observed, confirming that the molecular effects are recapitulated in vivo. At the systemic level, pungenin significantly reduced serum MDA and increased SOD and T-AOC levels, indicating an attenuation of oxidative stress. This antioxidative effect is consistent with the observed preservation of PTEN/AKT signaling, as excessive ROS is known to impair AKT phosphorylation through PTEN oxidation. Collectively, the in vitro gene expression, systemic biochemical profiles, and in vivo histological recovery (increased follicle density and dermal thickness) show a coherent, dose-related trend, supporting the interpretation that pungenin promotes hair regeneration through a combination of direct transcriptional regulation and indirect antioxidant protection.

While our findings provide a coherent mechanistic framework, several aspects warrant consideration in future studies. The primary cells used in this study were ORS cells rather than dermal papilla cells, so our conclusions primarily reflect epithelial responses. Additionally, the transcriptomic analysis was exploratory and will require functional validation of individual DEGs to fully establish causality. Addressing these points would further strengthen the evidence base for pungenin as a therapeutic candidate for AGA.

## 4. Materials and Methods

### 4.1. Primary Culture of Mouse Hair Follicle Cells

Primary hair follicle cells were isolated from the whisker pads of neonatal C57BL/6 mice between postnatal days 0 and 3. After sterilization in 75% ethanol, the excised tissues were rinsed with PBS containing 1% penicillin/streptomycin. Each hair follicle was gently dissected under a stereomicroscope (Nikon, Tokyo, Japan) and digested in a collagenase I/neutral protease II solution at 37 °C for 16–18 h. The suspended cells were sequentially passed through 200 μm and 100 μm cell strainers before centrifugation for 5 min at 300× *g*. The pelleted cells were collected into complete hair follicle culture medium, inoculated in culture dishes precoated with rat tail collagen, and grown at 37 °C with 5% CO_2_, exchanging culture medium at three-day intervals until 80% confluent. These cells were identified as outer root sheath (ORS) cells, as confirmed by CK-19 immunofluorescence staining, a specific marker for ORS cells in hair follicles.

### 4.2. Immunofluorescence Identification of CK-19 in Mouse Hair Follicle Cells

Early-passage primary mouse hair follicle cells (1 × 10^5^/mL) exhibiting stable growth were cultured on 15 mm glass coverslips in 24-well plates at 37 °C with 5% CO_2_ until reaching confluence. Cells were rinsed three times with PBS before fixation for 15 min in 4% paraformaldehyde. After an additional three PBS washes, residual buffer was removed, the cells were blocked for 30 min with normal goat serum at room temperature and treated overnight at 4 °C with an anti-CK-19 primary antibody diluted 1:100. After a further three washes in PBST, the coverlips were treated for 1 h at 20–37 °C with Cy3-conjugated goat anti-rabbit IgG (1:100) under light-protected conditions. After three additional PBST washes, nuclei were counterstained using DAPI for 5 min in the dark. Coverslips were subsequently washed four times with PBST, mounted using antifade mounting medium, and examined with a fluorescence microscope (Motic, Hong Kong, China).

### 4.3. Effects of Pungenin on Hair Follicle Cell Proliferation and Morphology

To assess how pungenin impacts cell proliferation, exponentially growing mouse hair follicle cells (5 × 10^4^/mL) were inoculated in 96-well plates and grown for 24 h under standard conditions before treatment with 0, 5, 10, 20, 40, or 80 μM pungenin for an additional 24 h. Cell proliferation was subsequently examined using the sulforhodamine B (SRB) assay. Briefly, 25 μL per well of 50% trichloroacetic acid (TCA) was introduced for 5 min to fix the cells, after which the plates were kept for 1 h at 4 °C. Following fixation, the plates underwent five washes with deionized water before air-drying at room temperature, staining for 30 min with 70 μL of SRB solution under light-protected conditions, and four washes with 1% acetic acid. After dissolving the protein-bound SRB in 100 μL of 10 mM unbuffered Tris base (pH 10.5), the plates were placed on an orbital shaker for 15 min, and absorbance at 540 nm was quantified via microplate reader (Molecular Devices Corporation, San Jose, CA, USA).

To examine the effects of pungenin on cell morphology, logarithmically growing hair-follicle cells (5 × 10^4^/mL) were inoculated into 6-well plates and grown for 24 h under standard conditions. Based on preliminary cytotoxicity experiments, the cells were assigned to four treatment groups: normal control, model (4 μM DHT) [30], finasteride (4 μM DHT plus 0.2 μM finasteride [31]), and pungenin (4 μM DHT plus 20 μM pungenin). Morphological alterations induced by the different treatments were monitored and photographed using phase-contrast microscopy(Motic, Hong Kong, China).

### 4.4. ELISAs

Exponentially growing hair follicle cells (5 × 10^4^/mL) were inoculated into 6-well plates and grown for 24 h under standard conditions. Following 24 h of treatment under the four experimental conditions (normal control, model, finasteride, and pungenin), cells were centrifuged (1000× g, 5 min, 4 °C). The *Srd5α2* contents of the supernatants were quantified using a commercially available mouse *Srd5α2* ELISA kit (Jiang Lai Biotechnology, Shanghai, China) as directed.

### 4.5. RNA Sequencing

Total RNA was extracted from cultured hair follicle cells using an RNA extraction kit (Jiang Lai Biotechnology, Shanghai, China) following the manufacturer’s instructions. RNA concentration and purity were determined spectrophotometrically at 260/280 nm. RNA libraries were prepared and sequenced using the Illumina NovaSeq platform (Shanghai Weihuan Biotechnology Co., Ltd., Shanghai, China). Raw sequencing reads were first subjected to quality assessment with FastQC (v0.11.8) and subsequent alignment to the mouse reference genome (mm10) using HISAT2 (v2.1.0). Gene expression was examined with FeatureCounts (v2.0.3), and differentially expressed genes (DEGs) were identified using DESeq2 (v1.22.1) in R, using the criteria of |log2FC| ≥ 1 and adjusted *p* < 0.05.

### 4.6. qPCR

qPCR was conducted on a QuantStudio 5 Real-Time PCR System (Thermo Fisher Scientific, Waltham, MA, USA) using SYBR Green Master Mix. Amplification consisted of an initial denaturation at 95 °C for 3 min, followed by 40 cycles of 95 °C for 15 s and 60 °C for 30 s. Relative expression was computed using the 2^−ΔΔCt^ method, using β-actin as reference.

### 4.7. Animal Model Experiments

Male C57BL/6 mice (SPF grade, 18–22 g) were obtained from Jinan Peng Yue Experimental Animal Breeding Co. (Jinan, China). Mice were housed in a SPF barrier facility under a 12 h light/12 h dark cycle, with ambient temperature maintained at 22 ± 2 °C and relative humidity at 50 ± 10%. Animals were kept in standard polysulfone cages (up to 5 mice per cage) with ad libitum access to steri-lized water and standard rodent chow. The whole animal experimental scheme was approved by the Experimental Animal Ethics Committee of Yantai University (Approval No.YDLL2025M027).

Following a 7-day acclimation period, the animals were anesthetized via intraperitoneal injection of sodium pentobarbital (40 mg/kg) before hair removal. Equal amounts of rosin and paraffin were heated until completely liquefied and then cooled to 45 °C. The molten mixture was evenly applied to a 2 × 3 cm^2^ area on the dorsal skin. After solidification, the hardened wax was removed to achieve complete depilation. Mice were allocated to five groups using a random number table, with seven animals in each group: One mouse per group was euthanized on day 0 for baseline skin histology. The remaining 6 mice per group completed the 21-day treatment. At the end of the study, 5 mice per group were randomly selected for serum biochemistry and dorsal skin histology; all 5 samples were included in the final analysis (*n* = 5 per group). No animals were excluded due to illness, injury, or outlier data. The groups were the control (Con), model (Mod), finasteride (Fin), low-dose pungenin (Pun-L), and high-dose pungenin (Pun-H) groups. Apart from those in the control group, all animals were given daily intraperitoneal injections of testosterone propionate (10 mg/kg in olive oil). Thirty minutes after injection, the dorsal skin of mice in the control and model groups was treated topically with olive oil alone, whereas animals in finasteride (positive control) group received 2% finasteride solution. Pungenin, previously isolated from the branches of *Picea wilsonii* Mast [26], was dissolved in olive oil to final concentrations of 5% and 10% (*w*/*v*) for topical administration to the low- and high-dose treatment groups, respectively.

At the end of the experiment (day 21), mice were deeply anesthetized with isoflurane. Blood was collected via retro-orbital puncture, and animals were then euthanized by cervical dislocation while fully anesthetized. Blood samples were collected from the eyes of each group of mice, and the serum was centrifuged for the detection and analysis of biochemical indicators. Then the full-thickness skin specimen measuring 2 × 3 cm^2^ was excised from the depilated dorsal region. Each tissue sample was divided into two portions, with fixation of one portion in 4% paraformaldehyde for histological analysis, while the other was immediately placed in liquid nitrogen before maintenance at −80 °C for subsequent qPCR analyses.

Throughout the experiment, animals were monitored daily for signs of distress, body weight loss, and skin irritation; no predefined humane endpoints were met, and no animals required early euthanasia. All procedures complied with the guidelines of the Experimental Animal Ethics Committee of Yantai University.

### 4.8. Plasma Biochemical Analyses

At the conclusion of the 21-day treatment period, blood samples were obtained from the retro-orbital sinus and centrifuged (3500 rpm, 10 min, 4 °C) to separate the sera. Total antioxidant capacity (T-AOC), malondialdehyde (MDA) concentration, superoxide dismutase (SOD) activity, and *Srd5α2* enzymatic activity were determined using commercially available assay kits as directed.

### 4.9. Hematoxylin–Eosin (HE) Staining

To evaluate skin architecture and hair follicle regeneration, dorsal skin samples were harvested on days 0 and day 21 following depilation. Following fixation for 24 h in 4% paraformaldehyde and subsequent paraffin embedding, the tissue samples were sectioned (5 μm) and stained with hematoxylin and eosin (H&E), after which skin thickness and hair follicle density were quantitatively analyzed using ImageJ (v1.52). Histological assessments were performed by an investigator who was blinded to the group allocation.

### 4.10. Network Pharmacological Methods

#### 4.10.1. Target Prediction

SwissTargetPrediction (https://swisstargetprediction.ch/, accessed on 12 August 2026) predicts compound targets based on ligand similarity to known active compounds. SEA (Similarity Ensemble Approach, https://sea.bkslab.org/, accessed on 12 August 2026) identifies targets by comparing compound similarity to sets of ligands with known targets. PharmMapper (https://www.lilab-ecust.cn/pharmmapper/, accessed on 12 August 2026) employs pharmacophore mapping to identify potential targets by matching compound conformations to target protein binding sites. The combined use of these three complementary approaches improves the coverage and reliability of target prediction for pungenin.

The chemical structure and SMILES notation for pungenin were retrieved from PubChem (https://pubchem.ncbi.nlm.nih.gov/, accessed on 12 August 2026). Potential targets of pungenin were predicted using SwissTargetPrediction, SEA, and PharmMapper. The predicted target lists from the three platforms were integrated, and duplicate entries were removed.

#### 4.10.2. AGA-Related Target Identification

Disease-associated targets for AGA were retrieved using “androgenetic alopecia” as the search term in the GeneCards (https://www.genecards.org/, accessed on 12 August 2026), OMIM (https://www.omim.org/, accessed on 12 August 2026), HERB (http://herb.ac.cn/, accessed on 12 August 2026), and DrugBank databases (https://go.drugbank.com/, accessed on 12 August 2026). The resulting datasets were integrated, and duplicate targets were eliminated.

#### 4.10.3. Identification of Overlapping Targets Between Pungenin and AGA

Predicted pungenin targets were intersected with AGA-associated targets to identify shared genes. A Venn diagram was employed when visualizing the overlap, and the intersecting genes were considered candidate therapeutic targets through which pungenin may exert anti-AGA effects.

#### 4.10.4. PPI Network and Identification of Core Targets

The common targets identified between pungenin and AGA were used for construction of a protein–protein (PPI) network using STRING (https://cn.string-db.org/, accessed on 12 August 2026). Key hub genes potentially involved in the therapeutic mechanism of pungenin were identified [32].

#### 4.10.5. GO and KEGG Enrichment Analyses

The overlapping targets shared by pungenin and AGA were imported into the Metascape (https://metascape.org/gp/index.html#/main/step1, accessed on 12 August 2026) platform to perform GO and KEGG functional analyses. The enrichment results were subsequently visualized graphically [33].

### 4.11. Statistical Analysis

All experiments were conducted independently in triplicate, and quantitative data are presented as the mean ± SD. Data were analyzed using GraphPad Prism 8.3.0. Multiple groups were compared using one-way ANOVAs followed by Tukey’s multiple-comparison test, while two groups were analyzed using *t*-tests. Data distributions were assessed using the Shapiro-Wilk test. *p* < 0.05 was considered statistically significant.

## 5. Conclusions

In conclusion, the present study demonstrates that pungenin promotes hair regeneration in a DHT-induced mouse model of AGA through multiple complementary mechanisms, including inhibition of *Srd5α2*, attenuation of oxidative stress, and transcriptional regulation of the PI3K/AKT/FoxO/mTOR axis [26,34]. The modulation of these core signaling pathways, particularly those governing cell survival and follicular cycling, supports a multi-targeted mechanism of action. Together, these results offer both mechanistic insight and experimental evidence supporting the continued development of *Picea wilsonii* Mast. and its bioactive constituent pungenin as promising therapeutic candidates for the treatment of androgenetic alopecia.

## Data Availability

The original contributions presented in this study are included in the article. Further inquiries can be directed to the corresponding author.

## Abbreviations

The following abbreviations are used in this manuscript:

AGA	Androgenetic alopecia
DEGs	Differentially expressed genes
DHT	Dihydrotestosterone
SPF	Specific pathogen-free
DPCs	Dermal papilla cells
H&E	Hematoxylin and eosin
MDA	Malondialdehyde
PPI	Protein–protein
Pun	Pungenin
ROS	Reactive oxygen species
*Srd5α2*	Type II 5α-reductase
SOD	Superoxide
SRB	the sulforhodamine B
T-AOC	Total antioxidant capacity
TCA	Trichloroacetic acid
